# Rethinking creative research methods in response to COVID-19: Creating a remote research kit

**DOI:** 10.1386/jaah_00078_1

**Published:** 2021-12-01

**Authors:** Rebecka Fleetwood-Smith

**Affiliations:** Department of History, School of Humanities, University of Bristol, 11 Woodland Road, Bristol, BS8 1TB, UK

**Keywords:** creative research methods, art, design, health, hospitals, healthcare environments, senses

## Abstract

The ‘Sensing Spaces of Healthcare: Rethinking the NHS Hospital’ project involves working with National Health Service (NHS) staff, patients and visitors to explore their experiences of hospital environments. Over the course of the project, creative approaches centred on art-based and design-led practices are employed to research people’s experiences. Such approaches often involve working closely with participants during sessions. As COVID-19 infection control measures have affected in-person research, it has been necessary to develop and adopt alternative low-contact approaches. This article presents the development of a remote creative research kit designed to be used without a researcher/practitioner present. The kit has been developed through work with creative practitioners, hospital arts organizations, patient and public contributors and learning from public engagement events. The remote creative research kit has led to rethinking and reimagining the ways in which such approaches may be of benefit more broadly in healthcare settings.

## Introducing the Sensing Spaces of Healthcare Project

Sensing Spaces of Healthcare is an ongoing UK Research & Innovation (UKRI) funded research project (2020–24) carried out in partnership with the hospital arts organizations Fresh Arts (North Bristol NHS Trust) and GOSH Arts (Great Ormond Street Hospital, London). The project involves exploring the sensory past, present and future of NHS (National Health Service) hospitals in England to identify ways in which specific hospital environments may be enhanced. The notion that the design of healthcare settings contribute to the health and well-being of those within the environments is widely accepted (see, e.g. All-Party Parliamentary Group on Arts Health and Wellbeing 2017; Anåker et al. 2017; Francis et al. 1999; Francis and Glanville 2001; Ulrich 1997; Ulrich et al. 2020). Whilst the concept that healthcare environments should be healing or therapeutic spaces is commonly recognized, debates endure as to what makes a healing environment (see, e.g. Dellinger 2010; Douglas 2004). This project involves considering multisensory, affective and emotional experiences of particular hospital settings. Working with project partners (Fresh Arts and GOSH Arts), who use the arts to enhance healthcare environments, findings will inform a series of prototypes designed to improve the respective healthcare settings.

The Sensing Spaces of Healthcare project involves using a range of approaches, including historical and creative research methods. This article presents the development of a creative research method in the form of a remote research kit. The kit has been designed in response to restrictions on in-person contact brought in by the pandemic and will be used to carry out research with NHS staff and visitors remotely, to explore their experiences of hospital environments. Rather than present the results of using the kit, this article focuses on the process of designing and developing the kit. Before presenting the development process, I first discuss creative research methods and the opportunities that they afford when carrying out research in healthcare settings.

## Researching Health, Illness and Healthcare Settings Using Creative Approaches

Creative research methods are at the heart of the Sensing Spaces of Healthcare project, including the remote research kit discussed in this article. Creativity in research can be difficult to define, as research is inherently creative (Leavy 2015). The term ‘creative research methods’ is used here to refer to both art-based and design-led approaches, such as creating a collage or the use of objects designed for elicitation. Creative research methods can be useful in addressing complex research questions as they can elicit knowledge that traditional research methods do not (Pink 2015). For example, Sophie Woodward (2020: 69) notes that such creative methods can enable researchers to explore and understand ‘non-verbal, sensory, kinaesthetic, material and imaginary ways of knowing’. Similarly, Dawn Mannay (2015: 32) discusses the ways in which creative approaches can provide a gateway to ‘new destinations’ or new knowledge as participants and researchers engage in a process of de-familiarization. For example, Mannay (2015: 32) found that the use of collage, photography and map-making led to new understandings of ‘place and space’ that lay beyond preconceived ideas. The use of creative approaches can, Mannay (2015) argues, lead to greater understandings than a solely verbal approach to data collection, whilst also prompting new ways of thinking for both participants and researchers.

Creative approaches are increasingly used to explore and represent experiences of health and illness. For example, Bernie Carter and Karen Ford (2013) found that combining the use of drawing, photography, story-telling and discussions allowed children to express their experiences of health and illness in a variety of forms. Similarly, Sarah McNicol and Cathy Leamy (2020) worked with people with dementia and artists to support people with dementia to express their experiences. In a different approach, Hilary Moss and Desmond O’Neill (2019) worked with artists and people with dementia, to create artworks to represent individuals’ experiences. The artworks were informed by art-making sessions held with people with dementia, rehearsals, performances and bedside dance sessions, alongside observations and interviews. Moss and O’Neill (2019) posit that creative approaches can illuminate the lived experience of health and illness. In the case of their research, they argue that the emotional and impactful insights developed would not have been possible without the use of art-based research methods.

Creative approaches have similarly been used to explore people’s experiences of healthcare settings. For example, Sarah Pink et al. (2020) found that combining photography, video recording, building tours and interview techniques led them to knowledge that would otherwise have been ‘unknowable’, eliciting findings that conventional evaluation surveys do not. Similarly, Margo Annemans et al. (2012) gave participants a camera, pen, pencils and paper, and invited participants to document their hospital stay. Their creative approaches conceptually brought participants into the research, and enabled participants to document their complex spatial experiences of a hospital.

Drawing upon the use of creative research methods and the vast opportunities that creative approaches offer when exploring healthcare settings, this project sought to develop a series of creative research methods to explore the experiences of staff, patients and visitors in specific areas of the hospital.

## Rethinking the Project’s Creative Research Methods

To develop this project’s creative research methods, I had planned to work with Fresh Arts and GOSH Arts as existing participatory arts programmes. I was to adopt the role of an ‘apprentice’ (Ellingson 2017) learning from those who work, spend time, and are patients in the specific settings. Developing the creative research methods in this way was designed to result in project/site specific creative approaches, shaped in response to the respective participatory arts programmes, whilst drawing upon existing methods (see, e.g. Kara 2015; Leavy 2015; Mannay 2015; Woodward 2020). Due to the pandemic, it was not possible to work this way, and this led to rethinking the approach both in terms of the process and the types of methods developed. For example, infection control measures necessitated remote approaches to carrying out research, whereby research is not carried out in-person.

Below I detail how I turned to (1) the ways in which creative practitioners and participatory arts programmes pivoted their practice to deliver programmes using blended techniques and (2) the use of cultural/design probes in design-led research, to begin designing and developing the remote creative research kit.

### Phase 1. Exploring blended approaches: Participatory arts programmes

Due to the restrictions brought in by the pandemic, creative practitioners and cultural organizations were required to adapt their participatory arts programmes/practices. I spoke with practitioners (visual artists, musicians and sculptors) who work with Fresh Arts and GOSH Arts to understand how individuals were rethinking and adjusting their practice. These informal discussions were held with individuals who work with the hospital arts organizations, as I had planned to join their sessions at the respective sites. Discussions centred on individuals’ practices prior to the pandemic and adaptations that they were making or planning to make due to the relevant restrictions. Many practitioners discussed the importance of materiality in their work: for example, the use of carefully selected art materials when working with individuals, and the ways in which they replicated these processes through curating packs of arts materials that could be delivered remotely. In some instances, these packs were delivered and included written instructions; in other instances such packs were accompanied with videos hosted, for instance, via YouTube.

Alongside conversations with individuals, I looked to the vast array of resources created and shared by cultural organizations and resources in use by the NHS National Performance Advisory Group (NPAG) of Arts, Heritage and Design in Healthcare Network. Mostly, I explored how organizations were able to continue working in the NHS. This was an informal process, whereby I watched YouTube clips created by arts practitioners, explored written and visual resources, such as GOSH Arts’ (n.d.) downloadable resources, and explored how programmes were delivered. For example, the charity Art in Hospital (n.d.) responded to the pandemic by providing parcels of art materials, activities and project sheets to the projects, patients and residents that they normally work with. CW+ (2020), the charity of Chelsea and Westminster Hospital NHS Foundation Trust, launched its ‘Arts for All: Virtual Connections’ programme, which is a digital arts programme that features video-recorded content produced by creative practitioners. The content is designed to support the health and well-being of patients, staff and those isolating in the wider community.

The processes employed by organizations and individuals alike typically involved hybrid means of engaging with people. In response to COVID-19, I sought to adopt a similar approach when designing the Sensing Spaces of Healthcare project’s creative research methods. This led to exploring the use of remote approaches, which do not necessitate face-to-face research.

### Phase 2. Exploring remote approaches to research: Cultural/design probes

Alongside learning from the hybrid practices taking place ‘on the ground’ in hospital arts programmes, I conducted a literature review of cultural probes and the opportunities they might offer for remote research. Cultural probes are typically used in design-led research to gather qualitative data to explore experiences and perceptions (Woodward 2020). William Gaver et al. (1999) first designed probe kits when working remotely with participants across Europe. The probes, a series of designed objects, were created to ask open-ended questions, inviting participants to complete and respond to a varied set of activities. Probe kits often include items such as single-use cameras, audio-recording devices, and stationery (Mattelmäki 2005), alongside a set of instructions. Instructions support participant engagement: for example, participants may be asked to use a camera to take a specific set of photographs. This method has been used in a range of settings including healthcare, for instance, Vesa Jääskö and Tuuli Mattelmäki (2003) explored nurses’ views of their work community using a series of probes.

Despite the potential opportunities that cultural probes offer, Jayne Wallace et al. (2013: 3443) claim that the frequent use of ‘off the shelf stationery’ within such kits typically limits the ways in which participants engage and respond to the probes. Thus, they advocate the use of probes that are ‘directed towards the phenomenon under study’ (2013: 3443). They recommend a careful design and development process to ensure that probes meet the needs of the project whilst being accessible and engaging for participants. Wallace et al.’s (2013) considered process of designing to support participant engagement draws parallels with the hybrid methods of delivery employed by arts organizations. For example, Arts Development Company (n.d.) commissioned ‘Arts at Home’, which included the creation of art resources that were delivered by post and digitally to support engagement by vulnerable groups during the pandemic.

## Designing and Developing the Remote Creative Research Kit

Drawing upon the hybrid ways of working employed by arts organizations during the pandemic, and the use of cultural probes within design research, we developed a remote research kit for exploring the senses in hospital environments. The creation of the research kit was collaborative and so I use the term ‘we’ to reflect the multiple perspectives involved in developing and refining the kit. The following section details the processes involved in designing and developing the research kit and highlights how developing and testing the activities allowed us to understand how people may use and engage with such methods and the opportunities that these afford when exploring sensory and emotional experiences. I include images from the development workshops (included anonymously with permission) and draw upon my reflective journal, to illustrate the processes involved.

In order to develop a series of project-specific probes (Wallace et al. 2013), we considered the aims of the research and the processes involved when carrying out research in the NHS. The use of cameras and audio recording equipment, often used in cultural probe kits, is typically restricted within NHS settings due to ethical concerns such as confidentiality and anonymity. Rather than ask people to capture aspects of the setting, we therefore explored probes that stimulate imagination (Gaver et al. 1999).

Due to the sensory focus of this research, we first considered how engaging with different ‘things’ such as objects, materials and images can elicit different insights (Pink 2015; Woodward 2020). Although this project does not ascribe to the notion of the traditional five senses, or that the senses can be treated discretely or in isolation, we began to explore probes with different tactile, olfactory, spatial and auditory qualities. Initially it was anticipated that each probe would be a ‘thing’ selected for its specific qualities; however, as the project developed, we began exploring the use of probes that act as prompts, inviting people to create something. The probes were honed through development workshops held with project partners, NHS staff, patient and public contributors and the project’s advisory board members. Each development workshop involved working with a probe in isolation, which allowed us to develop each probe in detail and carefully focus on how individuals engaged with and used each probe.

## Hosting the Development Workshops

Our development workshops involved inviting individuals to take part in a creative workshop and work with us to explore how the probe (i.e. an activity) could be developed. The invitation explained that the workshops were for the purpose of method development and not for the purpose of research. Workshops were held with project partners and their wider teams, with NHS staff (invited by our project partners), with Great Ormond Street Hospital (GOSH) Young Persons’ Advisory Group for research (YPAG) and with members of our project advisory board. Six workshops were held with groups ranging from six to ten attendees. In some cases individuals took part in one session; in other cases individuals were involved in multiple sessions. When working with YPAG we worked closely with Deirdre Leyden (Patient and Public Involvement and Engagement [PPIE] Lead/GOSH YPAG facilitator) and GOSH Arts to facilitate the session. Each young person received an e-gift voucher to thank them for their contribution.

The development workshops adopted a blended way of working, much like those used by many arts organizations, which involved posting a kit of materials to individuals and then hosting a creative workshop live via Zoom. During the workshops we discussed the process of taking part and how we could improve or change the activity ahead of our work with research participants. Sending materials during the pandemic was a careful process: each person received their own sealed kit of materials, which had been quarantined for a period of no less than 72 hours prior to a workshop. We followed the University of Bristol’s guidelines for using Zoom when hosting the workshops. For example, only those registered were admitted to the passcode-protected sessions, and functions, such as screen sharing, were limited to the hosts. The following examples detail the intricacies involved in the workshops and the ways in which creating together identified areas for development.

### Example development workshop 1: ‘Collaging a Future Hospital’

The first probe developed was a collage activity. Collage involves using materials, images, patterns and colours to portray something. Collage, as an art-based form of inquiry, has been used as a reflective process, an elicitation tool and to conceptualize ideas (Butler-Kisber 2008). The approach is considered an accessible, engaging method (Butler-Kisber and Poldma 2010; Culshaw 2019; Vacchelli 2018). We developed a collage activity to explore how people think and feel about the hospital and how they may reimagine the hospital environment.

**Figure 1:**
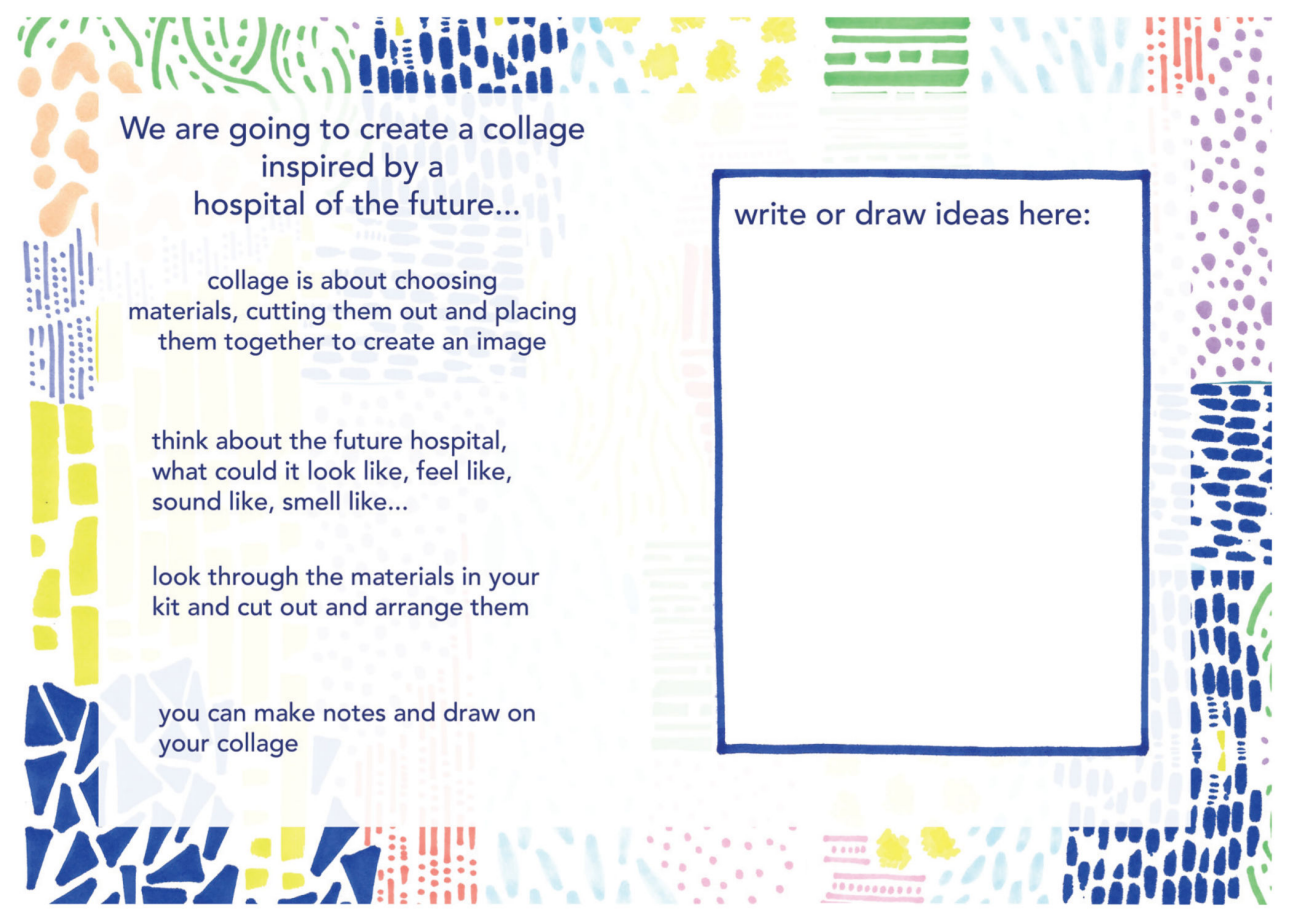
‘Collaging a Future Hospital’ booklet.

An initial development workshop, titled ‘Collaging a Future Hospital’, was held via Zoom with project partners and the facilitator of YPAG. This workshop was designed to explore the potential of the collage activity when working with children and young people. Prior to the workshop, individuals were sent a box of collage materials with an accompanying booklet that guided them through the process (see Figure 1).

At the beginning of the workshop, attendees were asked to spend a few minutes opening their collage pack and create their own collage ‘workspace’. This way of working drew upon how I typically work when leading creative sessions in-person. For instance, creating a space conducive to creativity is something that I would do through carefully curating the physical space; for example, rearranging furniture and displaying art materials to encourage intrigue. The collage box was designed to replicate processes involved when leading a creative workshop in-person. For example, materials were curated (see Figure 3) and grouped according to their colour, texture, pattern or type. Moreover, the collage booklet was carefully designed to be appealing and support engagement through the use of colour and pattern (see Figure 1).

In order to create their collage, attendees were invited to think about how they would want their future hospital to make them feel, for instance, calm, happy and inspired. They then explored their collage box, selecting materials that they associated with their chosen feeling(s). Using their selected materials, they created an abstract collage that represented their chosen feeling(s). Inspired by ‘draw, write and tell’ approaches (e.g. Angell et al. 2015), individuals wrote about their process of creating their collage, explained their choice of materials, and described how their collage represented their future hospital. For example, one workshop attendee explained that they used round shapes and soft materials to create a cocoon-like shape when thinking about a supportive environment.

**Figure 2:**
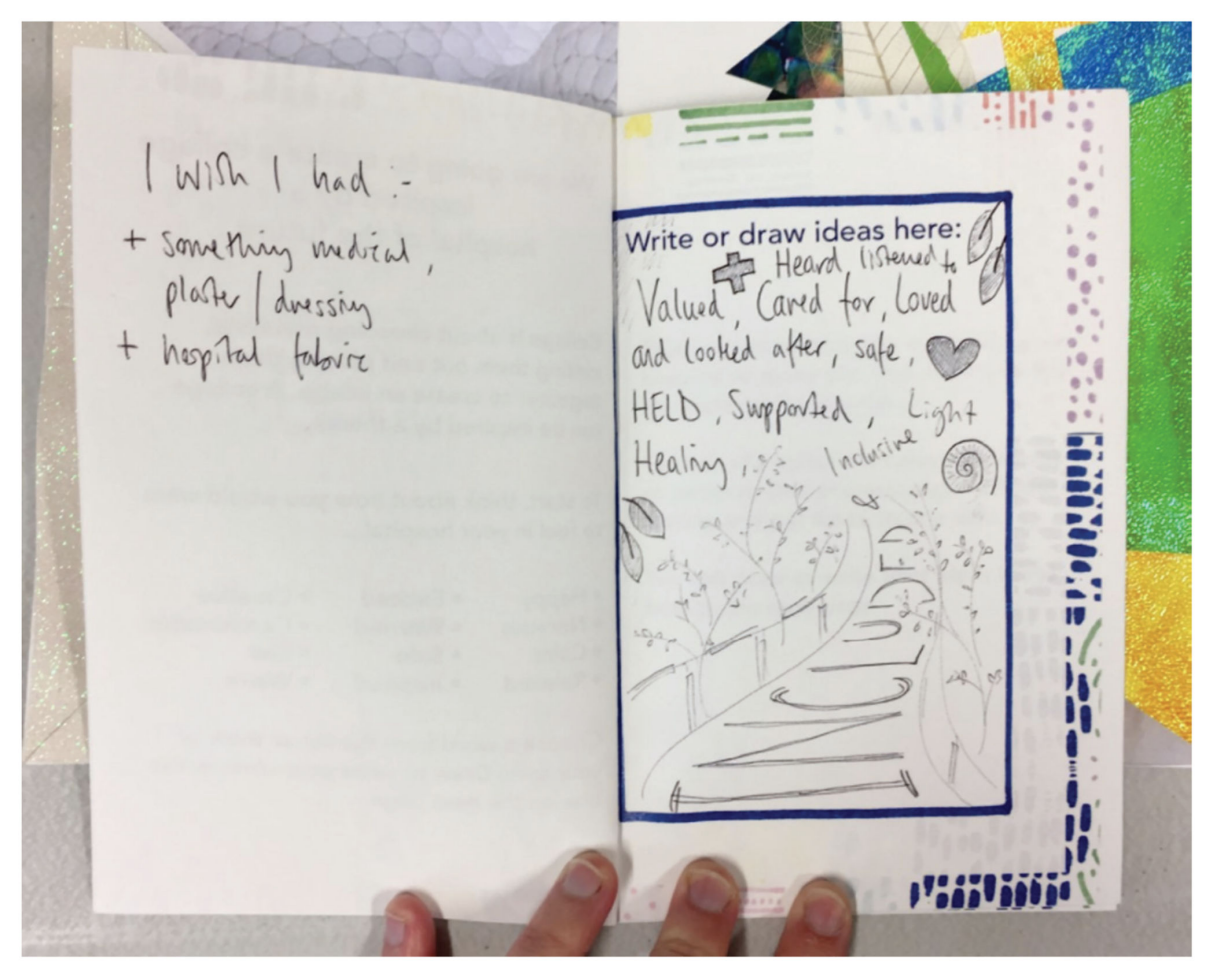
Recording development ideas in the collage booklet.

**Figure 3:**
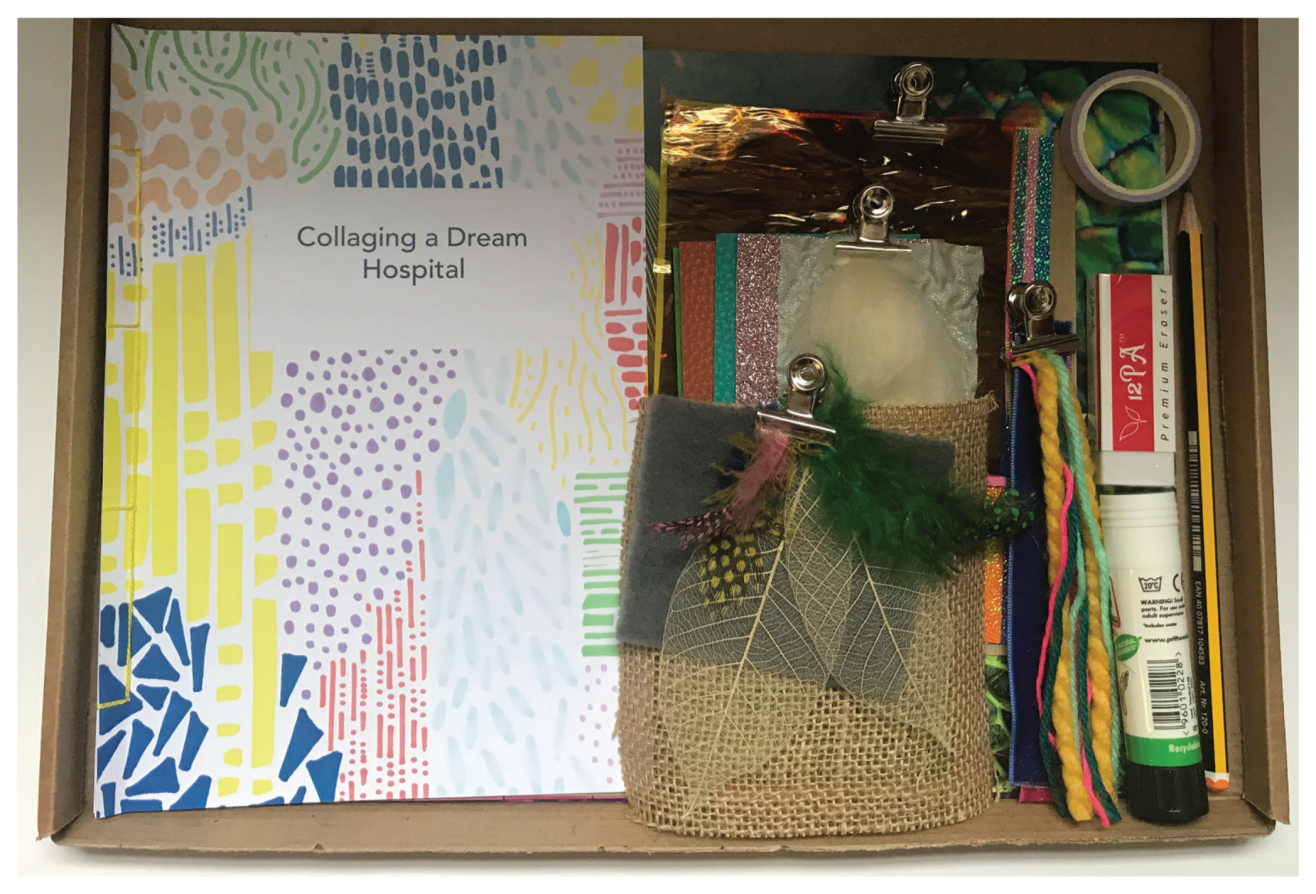
‘Collaging a Dream Hospital’ kit.

The process of creating collages led workshop attendees to think through the collage-making process and identify areas for improvement. Figure 2 depicts a list of materials that an attendee wished had been provided in the collage kit. Development discussions involved considering how the activity was presented, including, for instance, the title, activity instructions and the materials provided. For example, workshop attendees thought that the title ‘Collaging a Future Hospital’ promoted thinking about specific design features and thus may potentially limit creativity. The activity was therefore repositioned to become ‘Collaging a Dream Hospital’, to support exploratory, imaginative responses (see Figure 3).

‘Collaging a Dream Hospital’ was then used in a development workshop with YPAG members aged 11–17 years, which followed the same process as the initial workshop. Working with this group, we identified how the activity could be developed to work with young patients. For instance, the process of creating together allowed us to explore how each attendee worked at a different pace. Whilst some took time formulating their ideas, others were quick to create their collages, and this suggested an extension activity may be useful when working with young people. Working together also allowed us to identify further materials suitable for this group. For example, attendees suggested that stickers, templates, shapes and natural materials, such as seashells, may be useful and appealing to young patients.

### Example development workshop 2: ‘Reimagining Touch in the Hospital’

A further probe that we have developed involved thinking about touch in the hospital. Initially it was thought that this probe would be a series of material swatches. However, inspired by the recent use of ceramic objects as design probes (Zino et al. 2021), we began to explore alternative ways to think about exploring touch. This led to an informal conversation with artist and physiotherapist Nicola Lidstone, who suggested working with clay to explore touch. Fiona Buchanan’s (2015) observation that when working with clay the malleable material can allow people ‘to think through their hands’, as it involves kneading, cutting and moulding, connects closely with Tim Ingold’s extensive writing on thinking through making and the improvisational aspects of ‘form-making’ (Ingold 2013). For instance, Buchanan’s (2015) work with clay, involves asking people to explore their feelings using the material; Buchanan then considers the clay forms created to be metaphors for feelings. Moreover, Elaine Argyle and Gary Winship’s (2018) work demonstrates that the medium of clay offers a unique experience, whilst being accessible and flexible. The process of using clay to explore and think through ideas was of interest in this project, as we invite participants to consider their sensory, affective and emotional experiences of hospital environments.

**Figure 4:**
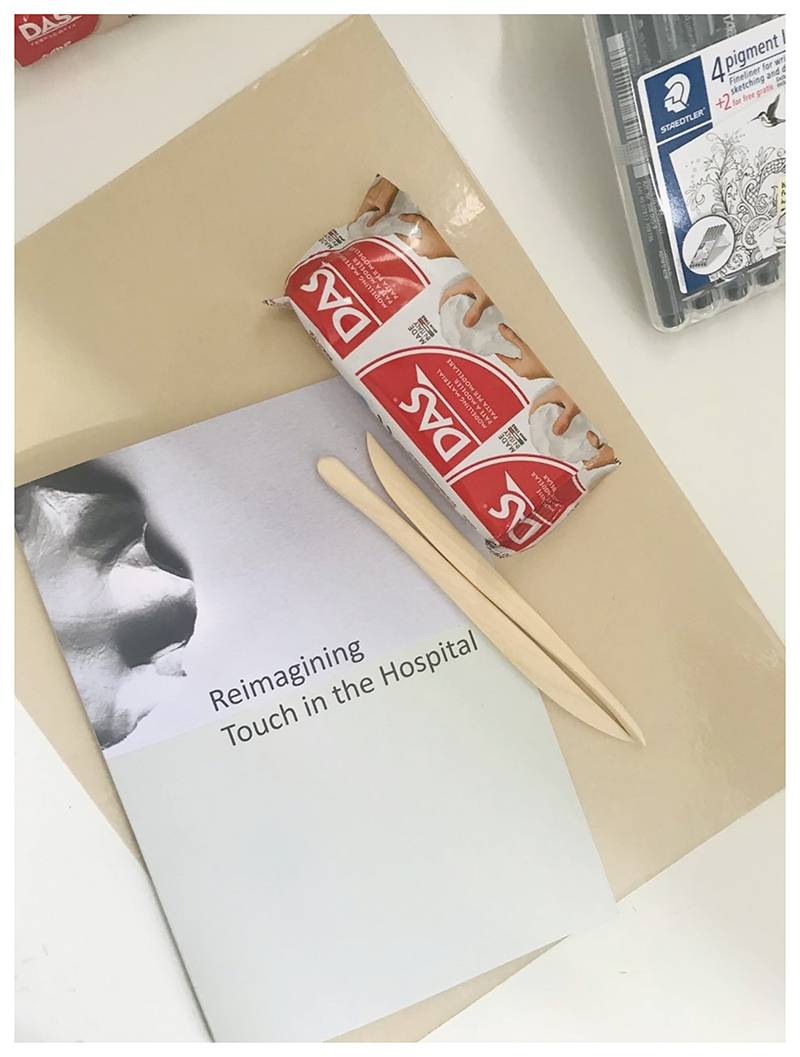
‘Reimagining Touch in the Hospital’ kit.

**Figure 5:**
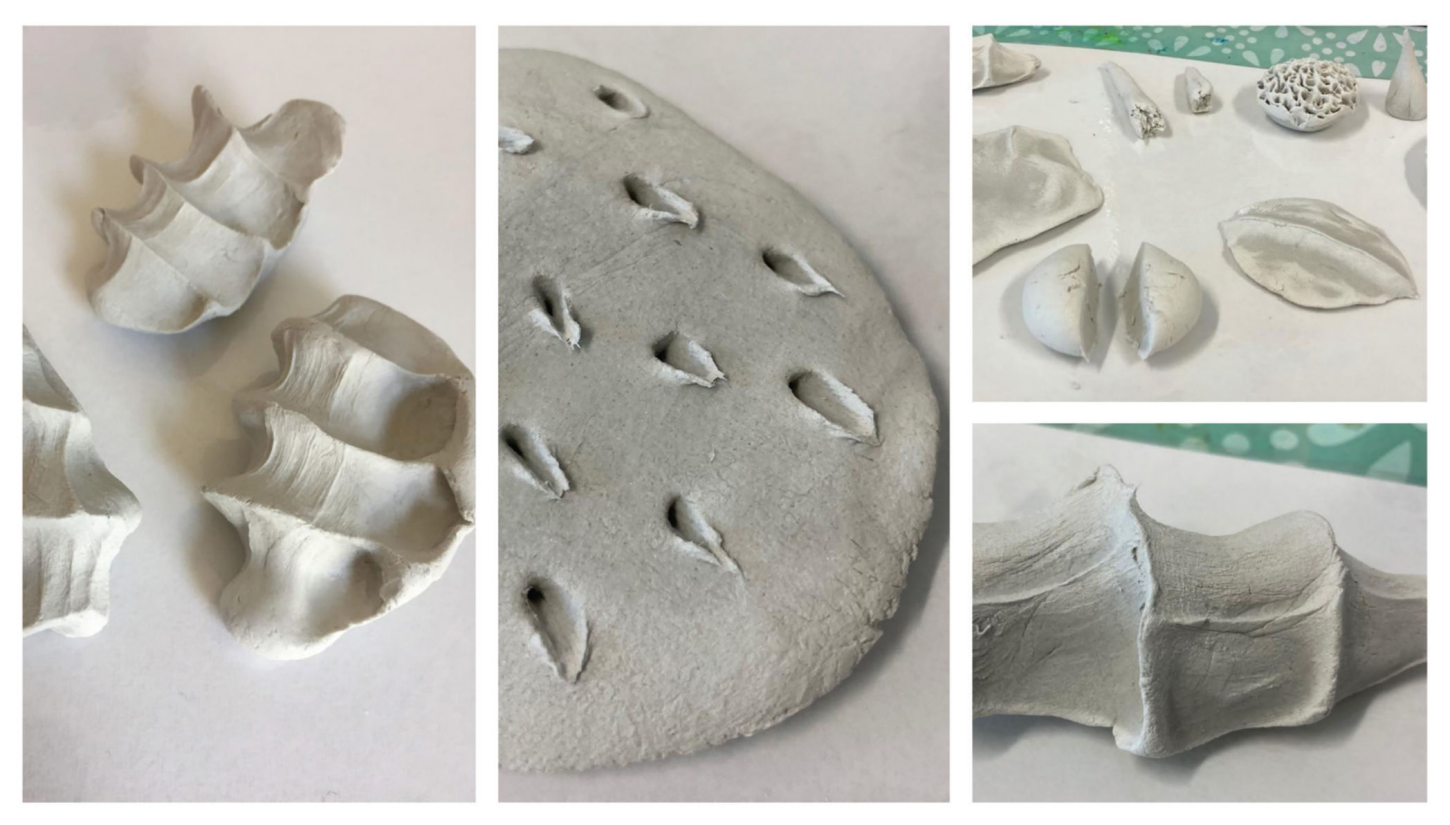
Workshop attendee’s clay forms – exploring multiple types of touch in the hospital, shared with permission.

We held a development workshop via Zoom with project partners and their wider teams to think through the process of working with clay. Prior to the workshop, individuals were sent a pack of materials (see Figure 4). As with the previous development sessions, I carefully designed the workshop materials to support engagement. Materials included: (1) a booklet that led attendees step-by-step through the activity and contained spaces where they could write their ideas; (2) a small pack of air-dry clay, selected as it is suitable for use at home; (3) lightweight wooden clay tools; and (4) a protective surface for attendees to create their clay forms on. Much like the ‘Collaging a Dream Hospital’, the process of working with clay involved inviting people to think imaginatively about touch in the hospital. Unlike the previous workshops, the session was split into the following stages: ‘warm-up’, ‘practice’ and ‘reflect’. These stages were designed to replicate processes that I typically use when leading workshops in-person. For the purpose of this activity, the ‘warm-up’ stage invited individuals to list as many different types of touch as they could within a few minutes. From there, individuals took part in the ‘practice’ stage, which involved spending up to twenty minutes creating a clay gesture that represented or reimagined touch in the hospital. (Figure 5 shows pieces created by two of the workshop attendees.) On creating their clay forms, individuals were asked to describe the pieces that they created (Buchanan 2015).

The ‘reflect’ stage involved two evaluation questions, developed with project consultant Jane Willis, that were designed to (1) record people’s thoughts about the activity and (2) explore whether the activity had prompted people to think differently about the hospital environment.

As with the collage workshops, insights regarding developing the activity occurred alongside the making process. For instance, attendees developed the title and instructions, focusing on how the activity was framed. They also recommended including an invitation within the instructions to encourage research participants to ‘be messy’ with the clay, to ensure that participants can create freely and are not concerned with producing a finished piece.

**Figure 6:**
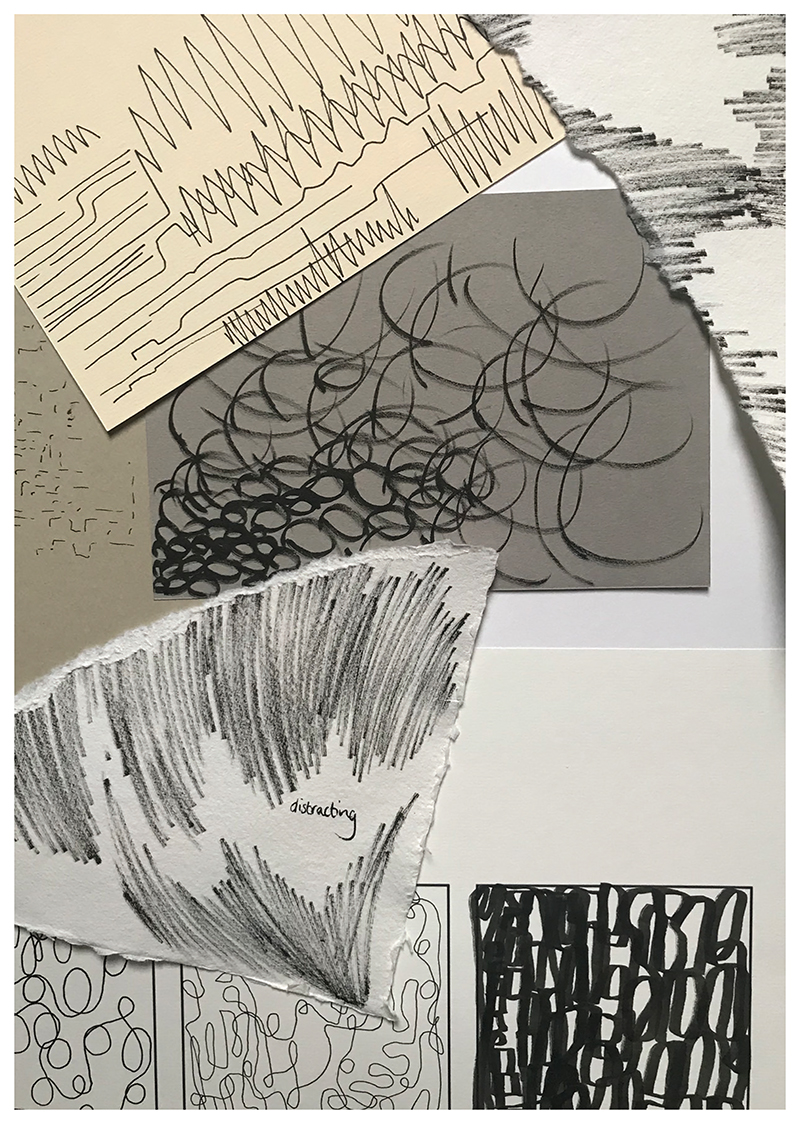
Marks I created in response to sounds of the hospital.

Following the processes presented here, we carried out further development workshops to refine each of the activities contained within the remote research kit. A further part of the design process occurred outside of the development workshops and drew upon informal learning from public engagement events. To share the project with the public, we have been commissioning creative practitioners, who work in health and social care settings, to run creative workshops for NHS staff during the pandemic. These events, although not methodological in focus, have provided interesting learning in terms of thinking through the project’s creative approaches. For example, I led a creative mark-making workshop for NHS staff titled ‘Listening to the Hospital’. The workshop involved listening to abstract sounds (recordings made at a hospital) from the Texture of Air project archive and inviting people to create marks in response to the sounds (see Figure 6).

In order to take part, individuals were sent a pack of specialist drawing materials, which included a range of fine liner pens and different surfaces: (varying textures, weights and sizes of paper/card). Attendees were then invited to take part in a creative workshop via Zoom. The different materials allowed attendees to explore spatial and auditory qualities through their use of materials and the marks that they made. Creating marks in response to the sounds also supported people to reconsider noise in the hospital and how certain sounds may make patients feel. Although the event was not a formal development workshop, it has since been developed to sit within the remote research kit.

## Sensing Spaces of Healthcare: Research Kit

Due to the iterative way of working, we consider the research kit presented here to be a prototype as this will continue to develop as we work with research participants. At present, the remote research kit contains five activities. Each activity is split into the following three stages:

1. Warm-up – a quick-fire stage which conceptually acts as a ‘route into’ the activity
2. Practice – the main activity, which invites participants to respond to or create something
3. Reflect – the final stage of the activity, which contains two short evaluation tasks.

The remote research kit contains the following activities:

1. Beyond the Hospital – this activity invites participants to think of some-where that they like to spend time, to select an item that reminds them of/or represents that place and respond to that item creatively
2. Collaging your Dream Hospital – this activity invites participants to explore what their dream hospital would feel like through abstract collaging
3. Listening to the Hospital – this activity invites participants to listen to abstract sounds from the hospital and make marks in response to these
4. Feeling the Hospital – this activity invites participants to explore what materials or touch between people could feel like in the hospital using clay
5. Mapping your Dream Hospital – this activity invites participants to think about the multisensory encounters that they would want to encounter in their dream hospital and map these on a floorplan.

**Figures 7 and 8:**
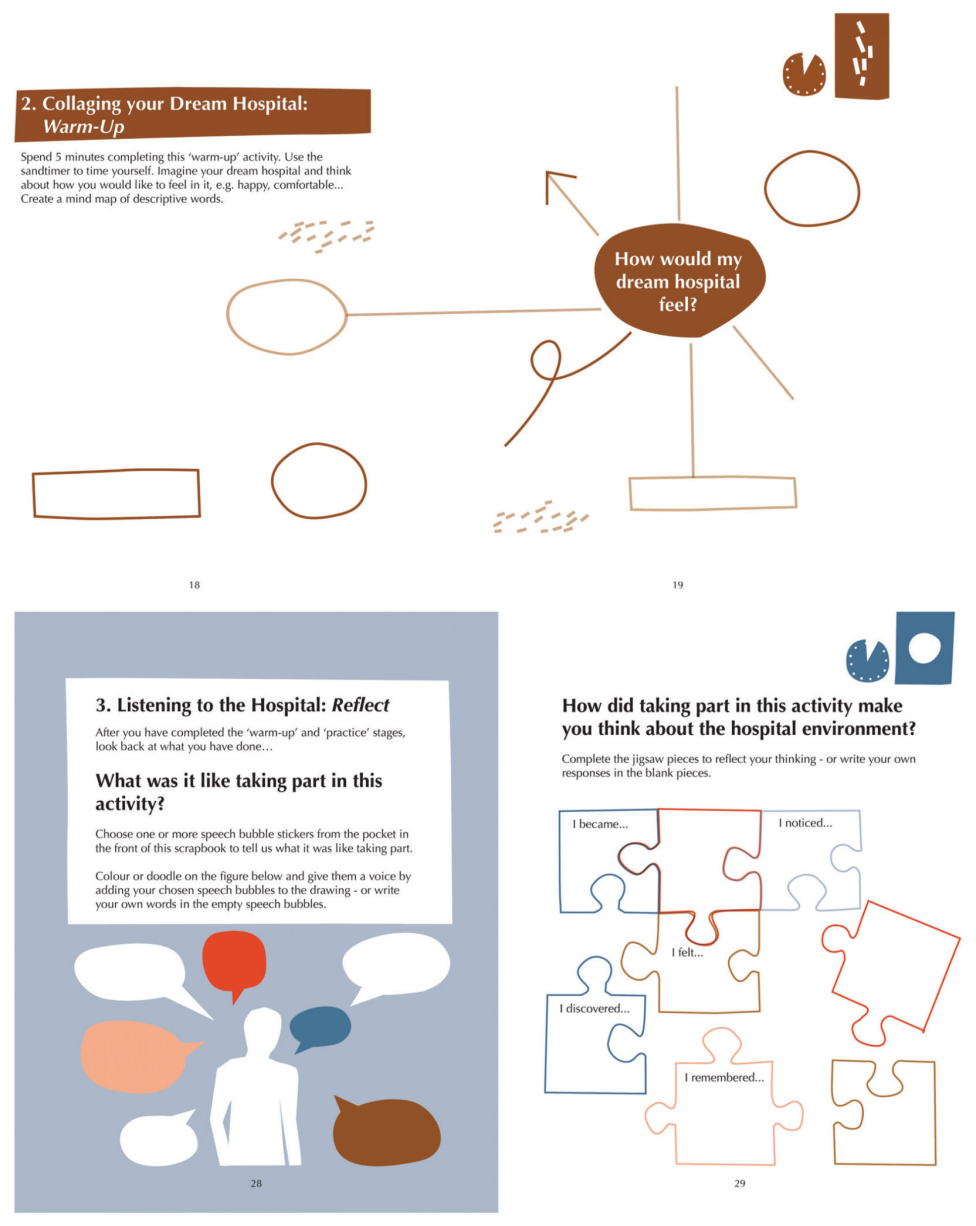
Selected pages from the activity book. Book design and illustration by Alex Higlett at Pirrip Press.

The activities and accompanying instructions are presented within an activity book contained in the kit (see Figures 7 and 8). The use of a book is inspired by Wallace et al.’s (2013) process of using written prompts to aid participants in scaffolding their ideas and is also informed by the use of solicited diaries in qualitative research (see Bartlett and Milligan 2015). The activity book was designed and illustrated by Alex Higlett at Pirrip Press to support participant engagement. We refined the design and layout of the book through some of our development workshops.

Participants will be invited to use the research kit in their own time, as opposed to using the kit in situ (for instance, when working at the hospital), and so the approach focuses on participants’ ability to reflect upon their experiences. Short videos accompany the kit and talk participants through each activity. On completing the kit, participants will be invited to a follow-up interview to describe and discuss their responses.

## Conclusion

The creation of the remote research kit has led to much unanticipated learning. Adapting the design and development process through a hybrid approach has led to considering the opportunities that working in this way affords. Firstly, that this process of developing creative research methods together is of value when designing methods to work with particular groups in certain settings. Secondly, the creativity and flexibility that the probes afford indicate the potential that the kit may have as a low-resource research method that could be of value in further areas of research, such as health service design. For example, the kit may complement existing toolkits, such as the Point of Care Foundation’s ‘Experience Based Co-Design Toolkit’ and it could be adapted for projects carried out in different health and social care settings. Conversations during development workshops also suggest that the kits could also be of value to different teams in the hospital. For example, healthcare planning and built environment teams who often work with staff and patients when planning changes to the hospital environment. Moreover, the kits could be adapted for different uses. For instance, YPAG members suggested that self-initiated creative activities could be given to inpatients to alleviate boredom in the hospital.

In this article, I have described the processes involved in rethinking a creative research method in light of the pandemic. Although our research findings are currently unknown, developing the remote research kit has enabled us to explore the opportunities offered by working through blended means, and indicated that remote kits might have much to offer in healthcare environments. Although this kit has been developed in response to the pandemic, remote and hybrid research methods offer specific opportunities for research and are of great value in their own right (Gaver et al. 1999; Mattelmäki 2005; Woodward 2020; Zino et al. 2021). These approaches can be especially relevant in specific settings such as healthcare environments as they offer flexible, adaptable participation and support participatory approaches to research. Although our kits have been designed as a research tool, they have also proven to be highly adaptable for different purposes. We hope, in time, to develop a version of our research kit that can be used by hospitals themselves to identify sensory challenges/opportunities in specific spaces. Overall, our development process has allowed us to explore the vast opportunities that blended approaches have to (1) the design and development of research methods and (2) the delivery and use of the research method itself. As this is an ongoing project, we will continue to test and develop these kits over coming years and will build on our findings about the specific opportunities they might offer.
